# Individualized physiology-based digital twin model for sports performance prediction: a reinterpretation of the Margaria–Morton model

**DOI:** 10.1038/s41598-024-56042-0

**Published:** 2024-03-05

**Authors:** Alice Boillet, Laurent A. Messonnier, Caroline Cohen

**Affiliations:** 1 LadHyX, UMR 7646 du CNRS, Ecole polytechnique, 91120 Palaiseau, France; 2 Université Savoie Mont Blanc, Laboratoire Interuniversitaire de Biologie de la Motricité, 73000 Chambéry, France; 3 Institut universitaire de France (IUF), 75231 Paris, France

**Keywords:** Metabolism, Bioenergetics, Computational models, Computer modelling, Metabolism, Bioenergetics, Computational models, Computer modelling

## Abstract

Performance in many racing sports depends on the ability of the athletes to produce and maintain the highest possible work i.e., the highest power for the duration of the race. To model this energy production in an individualized way, an adaptation and a reinterpretation (including a physiological meaning of parameters) of the three-component Margaria–Morton model were performed. The model is applied to the muscles involved in a given task. The introduction of physiological meanings was possible thanks to the measurement of physiological characteristics for a given athlete. A method for creating a digital twin was therefore proposed and applied for national-level cyclists. The twins thus created were validated by comparison with field performance, experimental observations, and literature data. Simulations of record times and 3-minute all-out tests were consistent with experimental data. Considering the literature, the model provided good estimates of the time course of muscle metabolite concentrations (e.g., lactate and phosphocreatine). It also simulated the behavior of oxygen kinetics at exercise onset and during recovery. This methodology has a wide range of applications, including prediction and optimization of the performance of individually modeled athletes.

## Introduction

Sports science encompasses a wide variety of disciplines which, among other goals, aim to optimize top level athlete performance. In racing sports, performing means minimizing the time (*T*) needed to cover a given distance (*D*). In this scheme, the time is linked to distance via the speed of the considered system (*v*) during the race as follows: $D = \int _{0}^{T} v(t) dt$. The competitor’s speed is related to the propulsive power output and to the resistive power (depending on discipline and equipment) via the equation of motion. The control variable of a given athlete involved in a given discipline/distance is the propulsive power delivered over time, more commonly referred to as the pacing strategy. Beyond a certain distance, the athlete cannot provide the maximal power throughout the race, like during a sprint, under threat of exhaustion before the end of the race. To determine what the optimal strategy should be, or in other words, how the athlete should choose the propulsive power over time to maximize performance but still be feasible, lots of race models have been developed^1,2^. A suitable one would allow the prediction of the ability to produce power at any time during the race. Our goal in this study was therefore to improve a theoretical model based on individual physiological characteristics and field performance data to make a predictive tool of individual athlete performances over races.

If we consider a combustion engine race, the maximal propulsive power production is the amount of fuel available/remaining in the tank, depending on the previous power produced. For a hybrid engine, there are two energy reservoirs to consider, plus a possible internal system for recharging the electric battery (replenishment during functioning). For a human-powered race, we also find constraints in terms of producible power over time, which are usually described in exercise science by i) the time to exhaustion at given constant powers-resulting in a time to exhaustion curve-, and ii) the power time-course during an all-out (typically 3-minute) trial^3–6^.

The power produced by humans for events lasting less than 15-20 minutes comes from the oxidative and non-oxidative (lactic and alactic) metabolic pathways^4,7^. By analogy with the tanks of a motorized vehicle, we can model power generation using flows out of fluid compartments. In this way, different hydraulic compartment models are used in sports science to predict the above-mentioned features characterizing the power-production capacities of an athlete. An ideal model would combine relative simplicity, high predictive accuracy, and make sense regarding the physiology, in that case, the energetic mechanisms underlying the power production. The existing ones are compromises between these three aspects including the following well-known ones that are nicely described in one of Dr Morton’s paper^8^. The critical power model (first introduced by Monod and Scherer^9^) only distinguishes between oxidative and non-oxidative energy sources. Although not always described as a hydraulic model, it works as one. The non-oxidative pathway corresponds to a reservoir of finite capacity (W’) but is theoretically limited by a maximal constant flow (maximal power). The oxidative pathway is associated with a reservoir of infinite size and limited maximum flow (corresponding to the critical power). The power produced by an individual corresponds to the outflow from the non-oxidative reservoir, itself being replenished by the oxidative reservoir^8^. Exhaustion is reached when the non-oxidative reservoir is emptied. This model is powerful in its simplicity but does not constrain in time the maximal power doable by an athlete nor does it model recovery/replenishment^8^. Margaria’s model^10^ distinguishes three (namely non-oxidative alactic, non-oxidative lactic, and oxidative) metabolic pathways, thus proposing a three-tank model. The three components aimed at providing a link between energy production and its physiological consequences (phosphagen breakdown, lactate formation and oxygen consumption, respectively). It does include a limitation of the power depending on the level of emptying of the non-oxidative reservoirs. However, Morton showed that the predictions of the model were not all realistic^11,12^. Therefore, Morton upgraded Margaria’s model by introducing elevation differences between reservoirs^13^, aiming to reproduce human physiology phenomena more accurately, namely what he defines as the “anaerobic threshold” or “onset of lactate production”. The resulting theoretical Morton–Margaria (M–M) model allows us to predict the time to exhaustion curve, as well as the time course of power for all-out exercises^14^. However, the published model relies on average phenomena and proposes parameters’ adjustments to correspond qualitatively, but not quantitatively, to the expected behavior. Therefore, this powerful theoretical model displays limitations in its practical use in its present form, especially to depict a specific individual.

Since its introduction, other works have used the M–M model and its variations to predict the time course of physiological parameters. In particular, the experimental time course of oxygen consumption ($\dot{V}O_2$) and its contribution to athletes’ power production have been compared to simulations obtained using the initial meanings of the model parameters. Discrepancies were observed between the simulated and the actual data^15,16^. Therefore, the authors themselves questioned the meaning of the variables or the way the models were adjusted to the studied subjects. Derivatives of the M–M model have also been used in conjunction with mechanical racing models to optimize racing strategies^17,18^. However, the majority of the works modeled the athletes using average values for the parameters, excluding individualization.

To overcome these two limitations (lack of predictive ability and individualization), Weigend et al.^15,19^ proposed to revisit the M–M model without giving physiological meanings to its parameters. The authors adjusted the parameters for a given athlete, solely based on the predictions of the athlete’s performance. Their approach appeared very efficient in terms of performance prediction. In particular, during intermittent exercises, their approach allowed more finite modeling of recovery/replenishment of compartment/component than other models previously proposed^20–23^. Weigend’s team work was promising and validated the interest in using a hydraulic model for performance prediction. Nevertheless, by construction, their model lost the ability to be interpreted in the light of physiological variables, turning the model into a sort of “black box”, where the only input variables were the athlete’s performance and no longer their physiological characteristics.

To overcome these challenges, we propose in the present study an approach based on the M–M model giving a physiological sense to the model, its geometrical parameters (heights and dimensions), and flow variables. In the Methods section, we first describe the working of the M–M model and the modifications we made to it. Then, we propose, from laboratory testing, the creation of a “digital twin” for a given athlete. In the Results and Discussion section, we compare simulations with field experiments and literature data.

## Methods

### A reinterpretation of the M–M model

#### What the model represents

**Figure 1:**
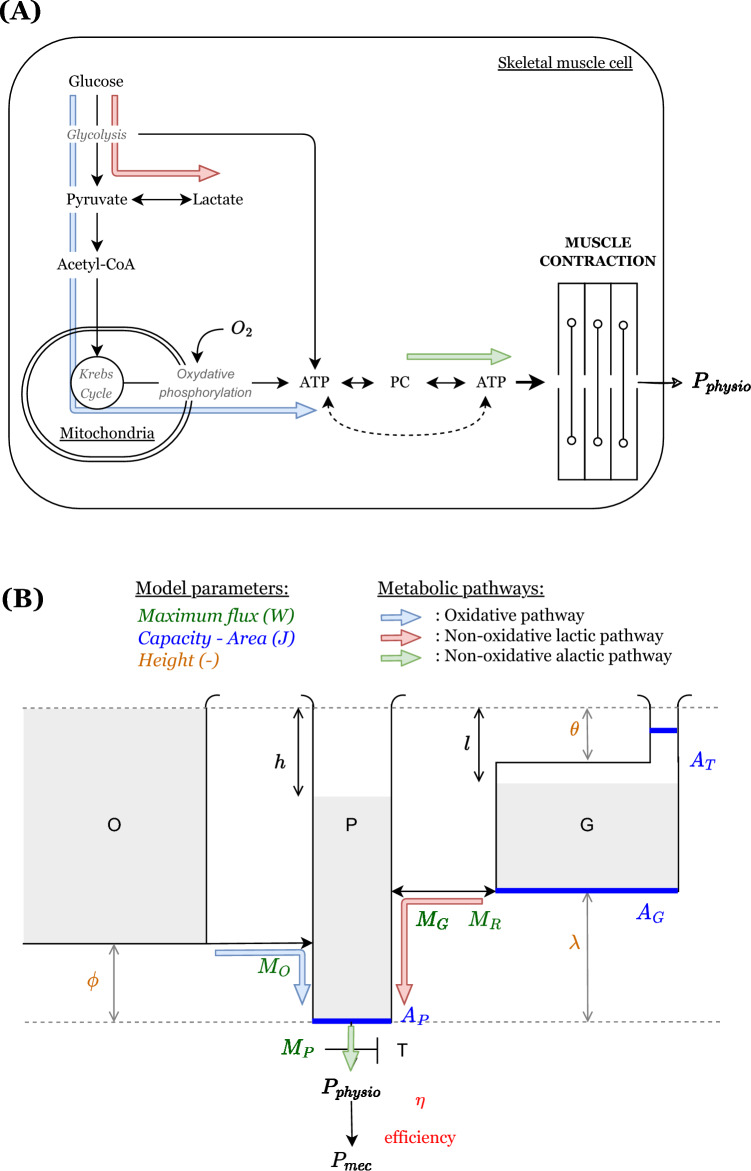
Modified M–M model—(**A**) metabolic pathway represented; (**B**) hydraulic model.

The hydraulic analog aims to describe the mechanical power production during exercise ($P_{mec}(t)$). For ease of understanding, a schematic diagram of the model is also provided in Fig. 1B. Table 1 summarizes the geometric parameters. The flow out (efflux) of the system is the physiological power ($P_{physio}(t)$) that the athlete has to generate to perform a given task. The effective mechanical power is linked to the physiological power by an efficiency factor $\eta$, $P_{mec}(t) = \eta P_{physio}(t)$. This parameter is considered constant, its meaning and determination will be clarified in more detail later. Each compartment of the system represents a source of power from the different energetic metabolism pathways involved in efforts of short to medium duration (1 min to $15-20$ min)^7^. It is composed of the three following compartments. The O compartment represents the energy coming from the oxidative pathway i.e., mitochondrial oxidative phosphorylation from carbohydrates and fat utilization (the latter being negligible in the context of physical exercises considered here: maximal or near maximal (> 80–85% of maximal oxygen uptake) and duration ($<15-20$ min) exercises. The O compartment is of infinite capacity. This means that, in the context of exercise intensity and duration considered, the oxidative energy source is theoretically infinite. The G compartment represents the energy coming from the non-oxidative glycolytic pathway (glycogenolysis and/or glycolysis not followed by mitochondrial oxidation). Efflux from this compartment could be assessed by the instantaneous net lactate accumulation rate or its oxygen equivalent. The P compartment represents the phosphagen pathway (energy from phosphocreatine—PC—and ADP breakdown to restore ATP which is *in fine* used as the ultimate energy substrate). G and P are of finite sizes. Efflux from O and G compartments feed compartment P. The fluid can flow out of the system through a tap *T* at the bottom of the P compartment with a fixed maximal diameter $M_P$. The total height of the system is 1. P is of height 1 and has a cross-sectional area of $A_P$. O is elevated by $\phi$ from the bottom of the system and is connected to P through a tube of diameter $M_O$. G is elevated by $\lambda$ from the bottom of the system and its main upper part is offset by $\theta$ from the top of the system. The cross-sectional area of this main part is $A_G$. It is connected to the outside (upper part of the system) through a thin capillary. The cross-sectional area of this tube is $A_T$. Morton considered it to be of negligible size ($A_T<< A_G$) and did not include it in his calculations. G is connected to P through a pipe of diameter $M_G$ in the G to P direction and of diameter $M_R$ in the P to G direction. These diameters and cross-sectional areas are all considered constant through time. The choice of parameters will be later justified (since their values can be adjusted thanks to the hereafter predictions and linked to athletes’ physiological characteristics). All compartments are subjected to the same pressure at their surface. Fluid flows are therefore governed by height differences between compartments. The emptying levels of P and G are denoted by *h* and *l*, respectively. Finally, it is assumed that the maximal power output is proportional to the level of fluid remaining in the main part of G (drain level, $A_G$ part). Thus the two governing equations of the system are:

1 $$\begin{aligned} \left\{ \begin{array}{ll} P_{physio}(t) = \dot{V}_P + D_{O \rightarrow P} + \dot{V}_G \\ P_{physio}(t) < P_{max}(t) = M_P\left( \frac{(1 -\lambda -l \textbf{1} _{l>\theta } - \theta \textbf{1} _{l\le \theta })}{1 -\lambda - \theta } \right) \end{array} \right. \end{aligned}$$

Where *V* stands for volume, $\dot{V}$ for volume variation with respect to time, and *D* for flow rate. $\textbf{1}$ is the indicator function, being equal to one when the condition stated as an indice is true, zero otherwise.

For a given athlete, Morton associated the changes in the compartments with physiological observations. More precisely, he mentioned that the efflux from O could be followed via whole body oxygen consumption ($\dot{V}O_2$) and the emptying of G could be approximated by blood lactate accumulation. However, the interventions of the different metabolic pathways and the production of mechanical power take place at the muscle level. We therefore propose a reinterpretation of the model by considering the active muscles instead of the whole organism. This interpretation is illustrated in Fig. 1. Following this line of reasoning, the modeled oxygen consumption is the muscular one, the G compartment emptying accounts for the net muscle lactate accumulation, and the modeled flux accounts for reactions at the muscles’ scale. If some intramuscular changes cannot be followed at the systemic level (e.g., PC levels), others can be followed but sometimes with a certain time lag. This is particularly the case between muscle and blood lactate concentrations^24^.

**Table 1:** Geometrical and flow parameters of the modified M–M Model (in the original M–M Model, $A_T$ is negligible).

$\phi$	Height of the bottom of O with respect to the bottom of P
$\lambda$	Height of the bottom of G with respect to the bottom of P
$\theta$	Depth of the top (of the larger section) of G with respect to the top of P
$M_O$	Maximum flow rate from O to P
$M_P$	Maximum flow rate out of P
$M_G$	Maximum flow rate from G to P
$M_R$	Maximum flow rate from P to G
$A_P$	Cross-sectional area of P
$A_G$	Cross-sectional area of the bottom part of G
$A_T$	Cross-sectional area of the small tube of G

#### Constant power modeling and physiological meanings

**Figure 2:**
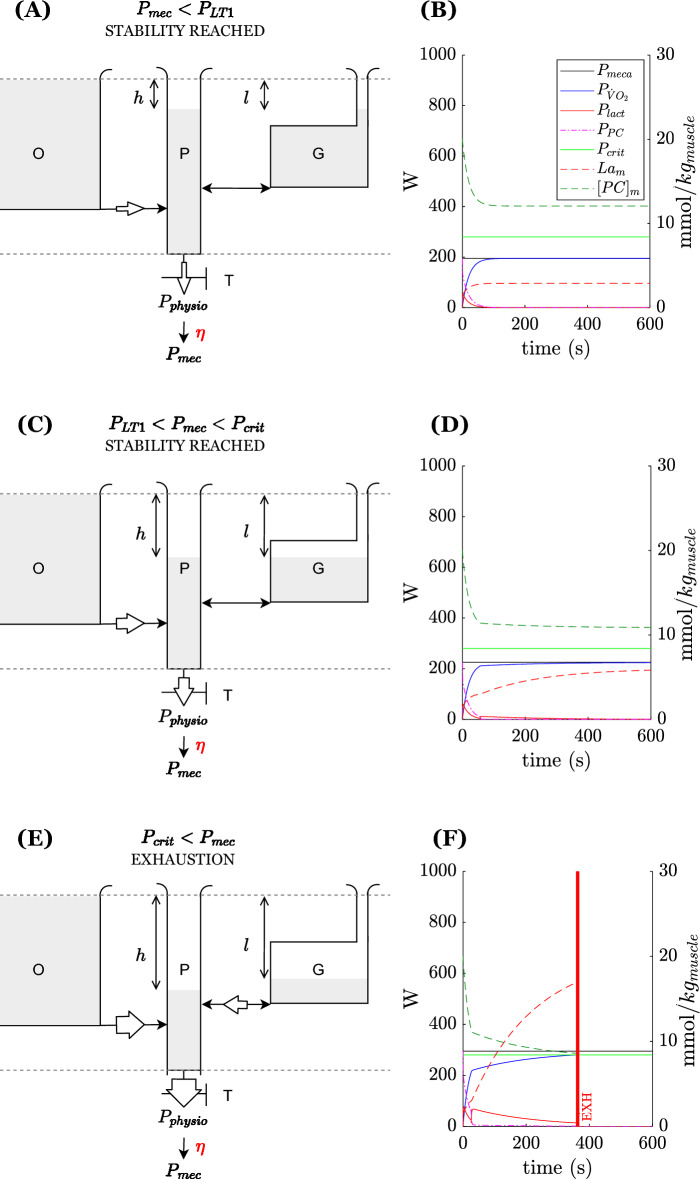
Simulations in the three intensity zones—(**A**) moderate (**B**) heavy (**C**) severe.

In the case of a constant-load exercise, Morton identified three intensity zones depending on the level of power imposed^13^. In the following lines, we used the terminology proposed by Beneke et al.^25^ to describe these zones. The behavior of the model for these three zones is reported in Fig. 2.

During moderate-intensity exercises (Fig. 2A,B), the tap T opens a little leading to a steady state associated with i) a slight decrease of level in P, ii) a constant flow from O to P, sufficient to cover the energy demand, and iii) no net flux from G to P. The reached steady state is such that $h<\theta$. In this regime, no “fatigue” is apparent, where fatigue corresponds in our model in any partial emptying of the main part of the G compartment. In other words, an athlete performing an exercise below this limit should always be able to reach their maximum power (maximal flux at T) observed in resting condition ($M_P$). Morton mentioned that when *h* reaches the $\theta$ value, that would correspond to an “anaerobic threshold”^14^ delineating the upper limit of this intensity zone. He remained unclear whether he was referring to the first or second lactate threshold^26^. However, given the above discussion based on the work of Beneke et al.^25^, it should correspond to the first lactate threshold (*LT*1). Using the model as stated by Morton, no increase in lactate concentration is predicted below $P_{LT1}$, which differs from the observations of Karlsson et al.^27^ and Chwalbinska-Monetta et al.^28^. Indeed, in these latter studies, *LT*1 marked an intensity beyond which a significant muscle lactate accumulation occurs. Below this threshold lactate concentration slightly increased but stabilized at a much lower value than above it. This small increase is coherent with the chemical interpretation of *LT*1 proposed by Mader et al.^29^. Because the M–M model predicts an unrealistic delay in the appearance of lactate in the muscle for a constant power at $P > P_{LT1}$, we modified it by introducing a non-negligible section $A_T$. This enables the model to better reflect the physiological reality since there are no such “purely aerobic” exercises. This can be visualized by referring to the Fig. 1A and the oxidative pathway. At $P<P_{LT1}$, an increase in the contribution of the oxidative pathway implies an increase in the concentrations of metabolic intermediates and a slight increase in the non-oxidative glycolytic (lactate) pathway. The chemical equilibrium means that muscle lactate concentration also increases since there is an intramuscular balance between pyruvate and lactate.

Secondly, for heavy-intensity exercise (Fig. 2C,D), the tap T is more open such that $h>\theta$. In this case, a flow from G to P occurs but is associated with an augmented flow from O to P, sufficiently to reach a new equilibrium. The upper limit of this intensity zone represents the maximum power for which an equilibrium/steady state of the system is still observable. Theoretically, this steady state and the associated power output could be maintained during an “infinite” duration. However, at present it is important to keep in mind that the model is built for exercises no longer than $15-20$ min. Monod and Scherrer^9^, defined the maximum rate that could be sustained by an athlete “for a very long time without fatigue” as the critical power ($P_{crit}$). Hill^30^, in his review of the concept also described the critical power as the asymptote of the power versus time to exhaustion relationship/curve. In the present model, as presented by Morton, the second threshold corresponds well to this notion of maintainable power for a long time: at least $15-20$ min. However, between the two thresholds, the athlete will already start to accumulate “fatigue” since they will no longer be able to produce at any time their maximum power measured in resting condition ($M_P$) because G will be partly drained. By construction, the upper limit of this heavy work rate zone is the power above which additional fatigue occurs (net flow from G to P, and decrease of the level in G) and below this power output, a recovery (replenishment of the G tank can take place, meaning a potential net flow from P to G if $l<h$). This is consistent with the notion of critical power and its role as a threshold between fatigue and recovery during intermittent effort^21,23,31^.

Finally, the severe work rate domain (Fig. 2D,E) corresponds to exercise intensities where the tap T is even more open. The augmentation in flow from O to P is not sufficient to fulfill energy demand. Flow from G to P is augmented and no equilibrium is reached during exercise. *l* continues to increase until exhaustion is reached. Constant power values in this zone can be used to establish a time-to-exhaustion curve.

In exercise physiology, these thresholds $P_{LT1}$ and $P_{crit}$ can be determined for a given athlete. Having clarified their meaning, we can envision an individualized model.

### Creation of a digital twin

The physiological meaning of the different model parameters can be used to build a “digital twin” of a given athlete for a given task. The model then becomes a predictive tool of performance and can be applied in optimizations without having to make the athlete undergo many time-consuming tests.

#### Required data

The different geometrical parameters of the model depicted in Table 1 can be determined via experimental measures. Both mechanical parameters ($P_{mec}(t)$) and the physiological responses of the athletes have to be monitored. To parameterize an athlete, the following characteristic values are required. For information purposes, we also indicate the protocols used for their measurements in our case (cyclists).

- Estimation of the muscle mass involved in the considered task ($m_{muscle}$)—Anthropometric measurements.
- Efficiency estimation $\eta$—Incremental test in the considered discipline with $\dot{V}O_{2}$ monitoring.
- Maximal oxygen consumption ($\dot{V}O_{2max}$)—Incremental test in the considered discipline with $\dot{V}O_{2}$ monitoring.
- Maximal power in resting condition (without fatigue)—All-out trial in the considered discipline (typically the first 6 seconds).
- Power output at *LT*1 ($P_{LT1}^{mec}$) and associated $\dot{V}O_{2}$ relative to $\dot{V}O_{2max}$ value: $\%\dot{V}O_{2max}=\frac{\dot{V}O_{2}}{\dot{V}O_{2max}}$ - Incremental test in the considered discipline with $\dot{V}O_{2}$ monitoring.
- Critical power $P_{crit}^{mec}$—3 minutes all-out trial.
- Maximal non-oxidative glycolytic (lactate) capacity for a non-intermittent effort above $P_{crit}^{mec}$, denoted $W_{non-ox}^{mec}$—3 minutes all-out trial.
- A power record curve of maximal effort with varying power output (e.g., a high-stakes competition).

We present the results for cyclists, therefore both incremental and 3-minute all-out tests were performed on a cycling ergometer (Excalibur Sport; Lode, Groningen, the Netherlands). The power record of maximal effort was obtained using instrumented pedals (Maxi Phyling, Palaiseau, France). Some parameters ($\phi$, $M_G$, $M_R$) will be indirectly computed via knowledge from the literature since their measurement in an athlete would be very invasive (e.g., determination of the time course of muscle lactate concentrations during exercise).

#### Muscle mass

The model is supposed to represent the active muscles of an athlete involved in a given motor task. This muscle mass ($m_{muscle}$) is the product of the mass of the athlete ($m_{athlete}$), their percentage of muscle mass ($\%_{muscle}$), and percentage of muscle mass involved in the considered exercise ($\%_{muscle \, for \, task}$): $m_{muscle} = m_{athlete}\cdot \%_{muscle}\cdot \%_{muscle \, for \, task}$.

#### Efficiency

The efficiency $\eta$, is the variable that links mechanical and physiological measures. In the present study, it is taken as constant, no matter the power requirement. This assumption constitutes a limitation of the model. Nevertheless, aware of this potential pitfall, we considered it constant as a first approximation^32,33^. This assumption could be challenged and discussed if the model predictions are not consistent. $\eta$ can be computed considering any constant power exercise for which all of the energy will be supplied by the oxidative pathway (i.e. inferior to $P_{crit}$). We chose $P_{LT1}$. We know the value of $\dot{V}O_2$ at this power, as well as the energy equivalent of oxygen ($C_1=20.9$ J/mL^7^). Efficiency can be calculated as the ratio between effective mechanical power at $P_{LT1}^{mec}$ and the metabolic energy generated by oxygen consumption. We therefore have :

2 $$\begin{aligned} \eta = \frac{P_{LT1}^{mec}}{\%\dot{V}O_{2max,LT1}\cdot \dot{V}O_{2max}\cdot C_1} \end{aligned}$$

#### Direct geometrical parameters

Then, we can set some parameters that are either directly measurable or directly assessable:

- $M_O$ is the maximum energy flux achievable by the aerobic pathway. Its physiological correspondence is therefore $\dot{V}O_{2max}$ (with energy conversion of $C_1$). $M_O = \dot{V}O_{2max} \cdot C_1$
- $M_P$ is the maximum energy flux achievable without any prior fatigue. It corresponds to the maximal power achievable by the athlete on the task, reconverted into physiological power $M_P = P_{max}^{mec}/\eta$
- $A_T$ indirectly represents the muscular lactate concentration at the first lactate threshold. Muscle biopsies at LT1 have shown it to be of $[La]_{muscle,LT1} = 3$ mmol/kg wet weight (vs. 1.5 mmol/kg wet weight at rest)^34^. Therefore $A_T = [La]_{muscle,LT1}/\theta \cdot m_{muscle}\cdot C_2$. With $C_2 = 100$ J/mmol being the conversion factor of accumulated lactate in muscle into Joules^7^.
- $A_P$ is linked to the maximum energy that could be extracted from the breakdown of ATP and PC present in the working muscle. ATP remains roughly constant during considered exercises^27^. Therefore $A_P$ is representative of the amount of PC stores in working muscles assuming the possibility of fully depleting those stores. $A_P = [PC]_{muscle,max} \cdot m_{muscle} \cdot C_3$. $C_3 = 43.3$ J/mmol being the conversion factor of PC into Joules^7^. $[PC]_{muscle,max} = 20$mmol/kg wet weight being the typical PC concentration in muscles^27,35^.

If $A_P$ and $A_T$ have been slightly incorrectly estimated, the error would be compensated by the later computation of $A_G$ (taking into account the whole non-oxidative energy that can be generated by a given athlete).

#### Relative heights

The geometrical parameters of the digital twin can be determined using simulations of constant power exercises. Indeed, such exercises have already been performed in the literature with measurements of intramuscular metabolites. We can therefore use these values to calibrate the model.

- $\phi$ is estimated using the magnitude of muscular PC depletion. Indeed, there is a relationship between the intensity of an exercise at constant power, expressed in $\%\dot{V}O_{2max}=\frac{\dot{V}O_{2}}{\dot{V}O_{2max}}$ and the level of PC depletion in the working muscles $[PC]_{muscle}$^27^. In the model, it is reflected by the linear relationship between the fluid level *h* in compartment P and the flow from O to P: $\%\dot{V}O_{2max} =\frac{h}{1-\phi }$ and $\Delta [PC]_{muscle} = h A_P /C_3/m_{muscle}$ leading to
  $$\begin{aligned} \phi = 1- \frac{\Delta [PC]_{muscle}m_{muscle}C_3}{A_P \%\dot{V}O_{2max}} \end{aligned}$$
  Using the data from Karlsson et al.^27^, we found $\phi = 0.30$ which is in good agreement with the value of 0.25 used by Benhcke^17^.
- $\theta$ and $\lambda$ are determined using the expression of the two power thresholds ($P_{LS1}$ and $P_{crit}$) in the model and associated percentages of $\dot{V}O_{2max}$ using Equation 1. Detailed calculations are available in the supplementary information.
  3 $$\begin{aligned} \left\{ \begin{array}{ll} \% {\dot{V}O_2}_{max,LT1} = \frac{{\dot{V}O_2}_{LT1}}{{\dot{V}O_2}_{max}}= \left( \frac{\theta }{1 - \phi }\right) = \alpha \\ \% {\dot{V}O_2}_{max,P_{crit}} = \frac{{\dot{V}O_2}_{P_{crit}}}{{\dot{V}O_2}_{max}} = \frac{(1-\lambda )}{(1-\phi ) + M_O/M_P(1-\theta - \lambda )} = \beta \end{array} \right. \end{aligned}$$

We get:

4 $$\begin{aligned} \left\{ \begin{array}{ll} \phi = 0.30 \\ \theta = \alpha*{(1 - \phi)} \\ \lambda = 1 - \frac{\theta \left( \frac{1}{\alpha }- \frac{M_O}{M_P}\right) }{\left( \frac{1}{\beta }- \frac{M_O}{M_P}\right) } \end{array} \right. \end{aligned}$$

#### G compartment adjustment

The remaining parameters characterizing G ($A_G$, $M_G$, $M_R$) are obtained from the predictions of the model for a 3-min all-out trial and from literature data as follows:

- $A_G$ is adjusted to account for $W_{non-ox}^{mec}$. Indeed, $W_{non-ox}^{mec}$ accounts for the level of draining in G determined by an all-out trial leading to $P_{crit}$. We used 3 min all-out trials because they have been shown to fulfill this requirement^6^:
  5 $$\begin{aligned} W_{non-ox}^{mec} / \eta = A_P\cdot l_{P_{crit}} + A_T\cdot \theta + A_G\cdot (l_{P_{crit}}-\theta) \end{aligned}$$
  And we get:
  6 $$\begin{aligned} A_G = \frac{W_{non-ox}^{mec}/\eta - A_P\cdot l_{P_{crit}} - A_T\cdot \theta }{l_{P_{crit}}-\theta} \end{aligned}$$
  Where $l_{P_{crit}}$ is obtained via Equation 1. Detailed calculations are available in the supplementary information.
- $M_G$ represents a maximal flux that is never reached in practice. It would require compartment P to be emptied instantaneously, which is neither possible in the model nor physiological conditions. It would indeed correspond to a situation where $[PC]_{muscle} = 0$ and $[La]_{muscle} = [La]_{muscle, rest}$ . Physiologically and chemically the phosphagens (phosphocreatine and ATP) stores are (in our domain of application) never fully depleted. They are always partially refilled with an increase in lactate accumulation within the myocytes (and with resulting $[La]_{muscle} > [La]_{muscle, rest}$). In simulations, to reach the observed maximal accumulation rate of muscular lactate (of $\approx 1-3$ mmol/kg wet weight,^36^ we had to impose a $M_G$ of 285 J/kg muscle mass involved in the task.
- $M_R$ is the maximal “recovery” rate i.e., the maximal elimination rate of lactate in the exercising muscles. It can be linked to the maximum reaction rate of the H-LDH (H-isoform of the lactate dehydrogenase) which catalyzes the oxidation of lactate into pyruvate. Wilkinson^37^ evaluated it to be linked to be 1/2.5 time the maximal reaction rate of the M-LDH (catalyzing in the reduction of pyruvate to lactate), leading to $M_R = M_G/2.5$. This value is coherent with other literature data^38^ in our case study where we create digital twins for athletes (national-level cyclists).

#### Example of resulting parameters

Now that all parameters are set, the simulation of a given effort $P_{mec}(t)$ is possible. To check the consistency of the model and to make possible adjustments, we use the recording of high-stake races (track cycling national and international competitions) where the effort is considered as a maximum one. We impose the effort on the digital twin. We expect the model to predict exhaustion just after the finish, or exhaustion when the power is increased by a few watts. If this is not the case, some of the limiting parameters have to be adjusted to achieve this result.

We use the data from four female national-level endurance track and road cyclists (mean age 22 yo, mean weight 64 kg, mean ${\dot{V}O_2}_{max}$ 4.1 L/min). This study was approved by the French National Ethics Committee, and all participants provided written informed consent for the study. All experiments were conducted in accordance with the Declaration of Helsinki guidelines (ClinicalTrial : NCT05314543). An example of the resulting parameters computed is available in Table 2.

**Table 2:** Geometrical and flow parameters of the modified M–M model for four cyclists (female, national-level).

Parameter	CYCLIST 1	CYCLIST 2	CYCLIST 3	CYCLIST 4
$\phi$	0.30	0.30	0.30	0.30
$\theta$	0.43	0.55	0.55	0.49
$\lambda$	0.38	0.23	0.28	0.34
$M_O$ (kJ/s)	1.34	1.45	1.39	1.58
$M_P$ (kJ/s)	4.48	5.52	4.84	4.23
$M_G$ (kJ/s)	9.15	10.89	8.96	6.13
$M_R$ (kJ/s)	3.66	4.36	3.59	2.45
$A_P$ (kJ)	27.79	33.10	27.24	27.95
$A_G$ (kJ)	320.3	370.0	497.8	486.7
$A_T$ (kJ)	11.27	10.37	8.53	9.88

### Computation methods

All simulations were performed with MATLAB R2021b software using forward Crank-Nicolson processes. The data from the literature were either directly extracted from the mentioned work or were extracted from publications’ figures using GRABIT function on MATLAB^39^.

## Results and discussion

In the following section, we will refer to the data from simulation as “Simulation” and from (field or laboratory) experiments or literature as “Experimental”.

### Athlete-specific simulation

#### Exercices at constant power in the different intensity zones

Exercises at constant power in the three identified zones were simulated for the four cyclists. For the sake of clarity, only the results i.e., time-course of variables of cyclist 1 in a time window of 10 minutes are reported in Fig. 2. The left panels ((A), (C), and (E)) depict the final state of the hydraulic model (steady-state for (A) and (C) or exhaustion for (E)). The right panels report the time-course of parameters hereafter described. Imposed $P_{mec}$ (solid dark line) and the contribution of this power supplied by the various metabolic pathways are given: $P_{\dot{V}O_2}$ (solid dark blue line) for the oxidative pathway, $P_{lact}$ (solid red line) for the non-oxidative lactic pathway and $P_{PC}$ (dash-point red line) for the non-oxidative alactic pathway. The theoretical time-course of muscle metabolite concentrations: $[PC]_{m}$ and $[La]_{m}$ in mmol/kg wet weight is also given (dashed green and red lines respectively). The $P_{crit}$ is also displayed (bright green solid line). To do this, we convert the time-course of *h* and *l* into muscle concentration using the energy equivalents of muscle metabolites and $m_{muscle}$. Finally, if the model predicts exhaustion (for $P_{mec} > P_{crit}$, (F)), the instant at which exhaustion (EXH) is predicted is illustrated by a vertical red line.

For a moderate-intensity exercise (Fig. 2A), an initial small part of the power is supplied by non-oxidative pathways (B). Rapidly, the oxidative pathway is sufficient to provide all the mechanical power required. $[PC]_{m}$ decreases before stabilizing around 12 mmol/kg wet weight in the chosen case. $[La]_{m}$ increases slightly before stabilizing around 2 mmol/kg wet weight for the simulated power ($95\% P_{LT1}$). The final state of the model is an equilibrium with $l\le \theta$, no more net flow is observed from G to P (B).

For a heavy-intensity exercise (Fig. 2C), a roughly similar behavior as for a moderate-intensity exercise is observed but $[PC]_{m}$ stabilizes at a lower value and $[La]_{m}$ at a higher one (respectively 11 and 6 mmol/kg wet weight for the chosen power ($80\% P_{crit}$). The final state of the model is a steady state with $\theta \le l \le l_{P_{crit}}$ (D). This means that no more net flow is observed from G to P at steady state.

For a severe-intensity exercise (Fig.‘2E), the imposed power is never fully provided by the oxidative source. $[PC]_{m}$ keeps decreasing and $[La]_{m}$ keeps increasing without reaching a stable value (F). Exhaustion is reached after a certain time (depending on the imposed power). The model just before exhaustion is such that there is still a net flow from G to P. At exhaustion the predicted $[PC]_m$ and $[La]_m$ are lower and higher than in the previous case, 9 and 18 mmol/kg wet weight respectively for the chosen power ($105\% P_{LT1}$).

In all three cases, the predicted behavior of the model and metabolites’ concentrations are coherent with previous literature data^40,41^.

#### All-out and time to exhaustion trials

**Figure 3:**
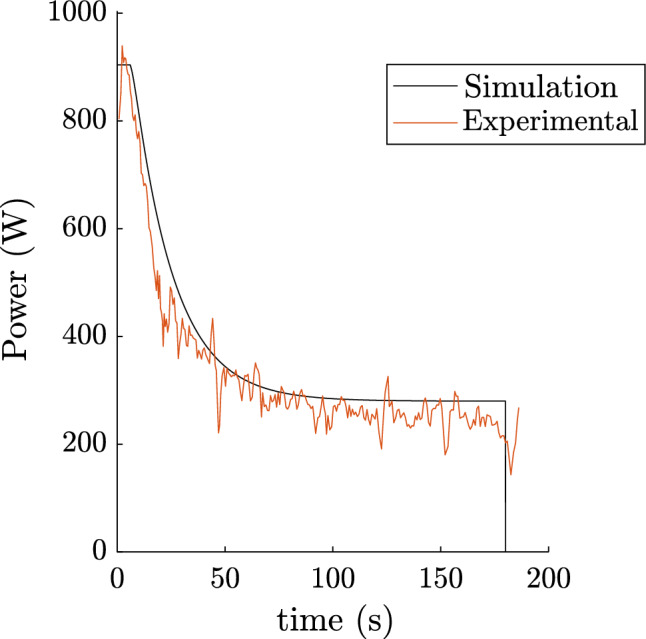
3 minutes all-out trial of Cyclist 1. Simulation output and experimental data.

**Figure 4:**
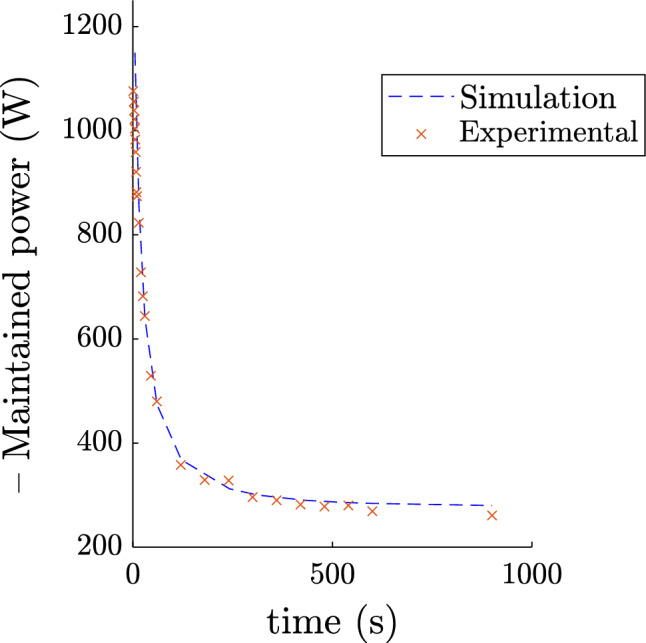
Time to exhaustion of Cyclist 1. Simulation outputs and experimental data.

The 3-minute all-out exercise can be simulated imposing at each instant: $P_{mec} = P_{max}\eta$, $P_{max}$ being given by Equation 1. This test is used to obtain the athlete’s critical power, non-oxidative capacity, and maximum power for the digital twin. However, we can ensure that the evolution of the simulation corresponds to the experimental data. The result for cyclist 1 can be seen in Fig. 3. By comparing the experimental and simulated all-out, we can see that the model does not take into account the intra-cycle variation in power. The power versus time to exhaustion relationship was also simulated, results are available in Fig. 4. The experimental data are from training power data records using the instrumented pedals. The simulations are in good agreement with the experimental data $RMSE = 52.7 W$ and $RMSE = 73.0 W$ for the all-out and time to exhaustion respectively (accounting for 15% and 16% respectively of average error rate). This confirms the predictive power of the proposed and developed model. The model seems to slightly overestimate the final power of the 3-minute all-out. Two points may explain this observation. Firstly, the efficiency term may vary in these fatigue conditions: after 3 minutes, when non-oxidative reserves are theoretically almost depleted, or at the end of close to 15-minute exhaustion exercises (accounting for the longer times of the time to exhaustion curve). Secondly, the theoretical power could be brought closer to the reality of these two tests by lowering the $P_{crit}$ entered in the model. However, if we do this, certain efforts made by athletes during competitions are no longer possible. Our aim is to model athletes at their maximum capacity, so we have chosen to keep this slight discrepancy in the presented data.

### Literature data

To check the consistency of predictions in terms of muscle metabolite concentration values and expected behavior, we used data from the literature. All shown simulations were done on different digital twins, again for the sake of clarity, only the data of Cyclist 1 are provided except for the last example.

#### Metabolic predictions

**Figure 5:**
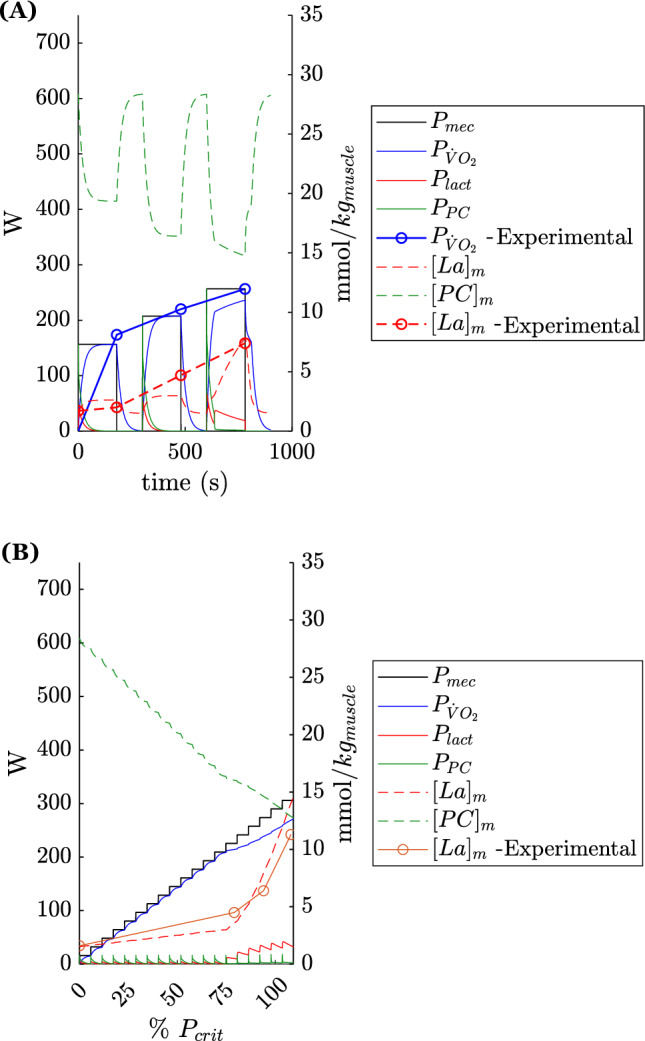
Metabolic prediction of the model versus literature data, (**A**) Chwalbinska-Moneta et al.^28^, (**B**) Green et al.^34^.

To test the validity of the predictions, we used published data of muscle metabolite concentrations obtained from muscle biopsies (during and/or at the end of the exercise) for given intensities (expressed in percentage of physiological thresholds detailed hereafter) for different subjects and were only the mean values were provided. We simulated the same exercises, adapting the power imposed on our models and cyclists to be at the same relative intensities for each digital twin. Resulting simulations compared to experimental data are presented in Fig. 5. The resulting parameters are displayed using the same color and lines as previously described. Experimental (discrete) data are represented using circles connected by lines, using the same color as their simulated counterparts.

In the study of Chwalbinska-Moneta et al.^28^, cyclists performed successively 3 minutes at 50 W below the power corresponding to the onset of blood lactate accumulation (OBLA), 3 minutes at the OBLA power, and 3 minutes at 50 W above OBLA power. Exercise bouts were performed 2 minutes apart. At the end of each power stage, muscle lactate value and percentage of $\dot{V}O_{2,max}$ reached were measured. Given that the OBLA power was reached at 60% of the $\dot{V}O_{2,max}$ power in their work, we used this data as a reference for our simulation. In other words, for our digital twins, we take the mechanical power at OBLA to be: $P_{OBLA} = 60 \% \eta M_O$, and increase/decrease on the various phases of the simulations to correspond to the detailed protocol. Results obtained from the simulations were compared to the experimental data, keeping in mind that Chwalbinska-Moneta et al. reported mean values. It is noteworthy that both in the paper of Chwalbinska-Moneta et al. and in the present study, endurance-trained subjects were studied. Results are available in Fig. 5A. Interestingly, the simulations provide very similar time-courses of variables than the experimental data, in both qualitative and quantitative terms. Even more importantly, predictions of muscle lactate and $\dot{V}O_2$ are concordant. A difference after the second exercise bout was observed between predicted muscle lactate values (around 5 mmol/kg wet weight) and that measured experimentally (around 3 mmol/kg wet weight). However, the discrepancy is relatively small and may be accounted by 1/ the experimental data is an average between different individuals contrary to our case, so it may be that the numerical twin presented here deviates from these averages. Indeed it is possible that the modeled athlete (national-level trained on intermittent-type efforts) has better recovery capabilities than the study subjects, which would explain the lower predicted muscle lactate by the model”.

In addition, we also attempted to reproduce the results and especially the time course of muscle lactate concentrations obtained during an incremental exercise by Green et al.^34^ Fig. 5B. In their study, the authors imposed an increment of 13.6 W per minute and took muscle biopsies at 0, 80, 95, 110% of the “anaerobic threshold” determined by the gaz exchange curve during the incremental test. As defined here, this threshold would roughly correspond to the second lactate threshold (LT2) or slightly lower. Although LT2 is different from critical power (they are closely correlated but LT2 is slightly lower than $P_{crit}$)^42,43^ we have adjusted the protocol as a percentage of critical power (considering $P_{crit} \approx P_{LT2}$). As it can be seen Fig. 5B, simulation and experimental data gave similar results in terms of both time course behavior and $[La]_m$ values.

#### Model behaviors

**Figure 6:**
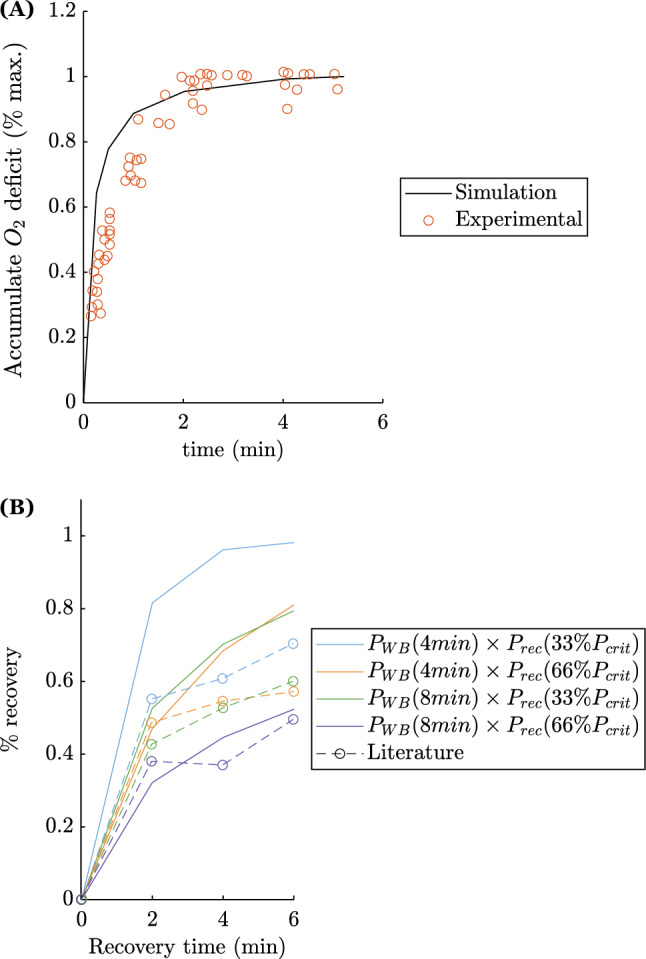
Behavioral prediction of the model vs. literature data, (**A**) Medbø et al.^44^, (**B**) Caen et al.^23^.

To further check the consistency of the model behaviors, additional comparisons with the literature were performed and can be seen in Fig. 6. Firstly, data from Medbø et al.^44^ were used. In this study, short very intensive exercises at constant power output were performed until exhaustion. For each exercise duration, accumulated oxygen deficit (AOD) was assessed. The same estimation can be done from the digital twin, calculating AOD via the amount of energy supplied by non-oxidative pathways. Results are provided in Fig. 6A. The parabolic behavior obtained experimentally by Medbø et al. was replicated in the simulations. This behavior means that the athletes cannot mobilize their maximum AOD for very short-duration exercise: typically below 2-3 minutes for Medbø et al.^44^ and 4 minutes for the model predictions. The slight difference between the study of Medbø et al. and the present study may come from the fact that an endurance-trained cyclist was used in our simulation. Indeed, it is not so obvious that the maximal AOD is reached in two or even three minutes in well-endurance-trained athletes. This behavior was expected given the model’s operating assumptions. The AOD can be associated with the level of emptying of G. Indeed, each time can be associated with a given exhaustion at a maximum maintainable power. This power is greater than $P_{crit}$, and can be maintained up to a certain level of emptying of compartment G according to Equation 1 such that: $\theta< l < l_{crit}$. As a result, the maximum AOD level is not reached. The shorter the time (the higher the corresponding power), the more the model will predict exhaustion for a lower discharge (lower *l*). Nevertheless, the consistency between the data of Medbø et al. and the present ones remains satisfactory.

Data from Caen et al. were also used^23^. In their study, eleven physical education students performed a constant-power exercise (at $P_{WB}$) until exhaustion (4 or 8 minutes), followed by a “recovery” phase at a submaximal percentage of $P_{crit}$, followed again by an exercise at exhaustion at $P_{WB}$. The same protocol was carried out on the four digital twins of our endurance-trained cyclists Table 2. The results for the four simulations are displayed in percentage recovery as defined in Caen’s work. Results are shown in Fig. 6B. Once again, the model predictions are consistent with literature data in terms of behavior. The difference in predicted values can be attributed to the non-athlete-specific parameterization of the $M_R$ recovery parameter and also partly to the different physical abilities of the subjects in the two studies.

Overall, the results obtained from simulations are consistent with literature data and good enough to validate the applicability of the model, keeping in mind its functioning assumptions. Discrepancies may also be attributed to (i) the fact that the experimental data in the literature are averages, whereas the simulation data presented here are intended to represent a single athlete, (ii) even as individuals, the subjects studied in the literature have a different background as the cyclists we used in or digital-twin examples.

## Limitations and perspectives

Although very promising, a few limitations to the model presented here must be pointed out. Some of the parameters were adjusted based on mean values found in the literature, whereas the essence of our work is to individualize as much as possible. As far as capacity values ($A_T$ and $A_P$) are concerned, we cannot easily envisage individualization, as this would require excessively invasive measurements of the athletes. On the other hand, for the recovery parameter $M_R$, it would be possible to individualize recovery capacity with appropriate tests. This type of test is not usually carried out under controlled conditions. Nevertheless, this is an area for our future work. Furthermore, the model only takes power as input, without taking into account strength or speed components. However, it has been shown, notably in cycling, that cadence has an impact on the ability to maintain a given power output^45,46^ (and therefore on fatigue). This model is therefore preferentially used in cases where the cadence at which the task is performed varies little, and where the tests used to parameterize the model have been carried out under similar speed conditions. Finally, for future applications, it is important to keep in mind that this model is designed to model efforts from one to 15-20 minutes.

## Conclusion

The reinterpretation of the M–M model, including a change in the model geometry with a non-negligible size of the capillary tube connecting the G compartment to the outside, and physiological interpretations of the model parameters, enables the creation of athlete-specific digital twins. Digital twins are a powerful tool in many aspects. It might afterward be useful in the context of athlete’s performance prediction and race or event strategy optimization. Its direct link with measurable physiological variables would also allow the prediction of potential gains in performance for changes in those physiological variables. This sensitivity analysis would be very interesting for the athletes and their coaches. In any of those applications, the model would also provide insight and valuable information about the origin of the produced power.

LaTeX formats citations and references automatically using the bibliography records in your .bib file, which you can edit via the project menu. Use the cite command for an inline citation, e.g.^47^.

For data citations of datasets uploaded to e.g. *figshare*, please use the [SPSVERBc1SPS] option in the bib entry to specify the platform and the link, as in the [SPSVERBc2SPS] example in the sample bibliography file.

### Supplementary Information

Supplementary Information.
