# Author Correction: Individualized physiology-based digital twin model for sports performance prediction: a reinterpretation of the Margaria–Morton model

**DOI:** 10.1038/s41598-024-66712-8

**Published:** 2024-07-10

**Authors:** Alice Boillet, Laurent A. Messonnier, Caroline Cohen

**Affiliations:** 1 LadHyX, UMR 7646 du CNRS, Ecole polytechnique, 91120 Palaiseau, France; 2 Université Savoie Mont Blanc, Laboratoire Interuniversitaire de Biologie de la Motricité, 73000 Chambéry, France; 3 Institut universitaire de France (IUF), 75231 Paris, France

Correction to: *Scientific Reports* 10.1038/s41598-024-56042-0, published online 05 March 2024

The original version of this Article contained a display error in Equations. 4, 5 and 6.

Equation 4

$$ \left\{ {\begin{array}{*{20}c}    {\phi  = 0.30}  \\    {\theta  = \alpha  - \phi }  \\    {\lambda  = 1 - \frac{{\theta (\frac{1}{\alpha } - \frac{{M_{O} }}{{M_{P} }})}}{{(\frac{1}{\beta } - \frac{{M_{O} }}{{M_{P} }})}}}  \\   \end{array} } \right. $$

now reads,

$$ \left\{ {\begin{array}{*{20}c}    {\phi  = 0.30}  \\    {\theta  = \alpha *(1 - \phi )}  \\    {\lambda  = 1 - \frac{{\theta (\frac{1}{\alpha } - \frac{{M_{O} }}{{M_{P} }})}}{{(\frac{1}{\beta } - \frac{{M_{O} }}{{M_{P} }})}}}  \\   \end{array} } \right. $$

Equation 5

$$\begin{array}{c}{W}_{non-ox}^{mec}/\eta ={A}_{P}\cdot {l}_{{P}_{crit}}+{A}_{T}\cdot \theta +{A}_{G}\cdot (1-\lambda -{l}_{{P}_{crit}})\end{array}$$

now reads,

$$\begin{array}{c}{W}_{non-ox}^{mec}/\eta ={A}_{P}\cdot {l}_{{P}_{crit}}+{A}_{T}\cdot \theta +{A}_{G}\cdot ({l}_{{P}_{crit}}-\theta )\end{array}$$

Equation 6

$$\begin{array}{c}{A}_{G}=\frac{{W}_{non-ox}^{mec}/\eta -{A}_{P}\cdot {l}_{{P}_{crit}}-{A}_{T}\cdot \theta }{1-\lambda -{l}_{{P}_{crit}}}\end{array}$$

now reads,

$$\begin{array}{c}{A}_{G}=\frac{{W}_{non-ox}^{mec}/\eta -{A}_{P}\cdot {l}_{{P}_{crit}}-{A}_{T}\cdot \theta }{{l}_{{P}_{crit} }-\theta }\end{array}$$

The original Article has been corrected.

