# Supplementary material for: Individualized physiology-based digital twin model for sports performance prediction: a reinterpretation of the Margaria–Morton model
Source: Sci Rep. 2024 Mar 5;14:5470. doi: 10.1038/s41598-024-56042-0 (PMC10915161; doi:10.1038/s41598-024-56042-0)
Supplement: Supplementary file 1 — Supplementary Information. [file 41598_2024_56042_MOESM1_ESM.pdf]

A Reinterpretation of Margaria-Morton Model towards an individualized physiological-based digital twin able to predict performance

Alice Boillet, Laurent A. Messonnier, and Caroline Cohen

Supplementary information

Here, we detail some of the calculations for the modified M-M model. These calculations are an adaptation of those presented in the description of Morton's original model[1–3], but extended to the modified M-M model we introduced in our article. We will use the same notations as described in the paper. The two governing equations of the system are:

$$\begin{cases} P_{physio}(t) = \dot{V}_P + D_{O\to P} + \dot{V}_G \\ P_{physio}(t) < P_{max}(t) = M_P\left(\frac{(1-\lambda - l\mathbf{1}_{l>\theta} - \theta\mathbf{1}_{l\le\theta})}{1-\lambda-\theta}\right) \end{cases} \tag{1}$$

Where V stands for volume, $\dot{V}$ for volume variation with respect to time, and D for flow rate. $\mathbf{1}$ is the indicator function.

From Equation 1 we can describe the behavior of the system in the three work-rate regions at constant power. We will express the kinetics of l (lactate accumulation) and $\dot{V}O_2$, as well as their values and associated P_{physio} at the thresholds between the regions.

Moderate work rate - Below LT1

We have $h < \theta$ and $A_T\frac{dl}{dt} = M_G\left(\frac{h-l}{1-\lambda}\right)$. Thus the surface of the G compartment remains in the capillary region. There is a linear relation between $\dot{V}O_2$ and $D_{O\to P}$: $\dot{V}O_2 = D_{O\to P}/C_1$. Therefore:

$$\begin{aligned} P_{physio} &= D_{O\to P} + \dot{V}_P + +\dot{V}_G \\ &= M_O\left(\frac{h}{1-\phi}\right) + A_P\frac{dh}{dt} + A_T\frac{dl}{dt} \end{aligned}$$

Similarly with the classical Morton model, one can get a second-order differential equation on either l or h.

$$a\frac{d^2l}{dt^2} + b\frac{dl}{dt} + cl = P_{physio}, \qquad \text{with:} \begin{cases} a = \frac{A_PA_G}{M_G}(1-\lambda) \\ b = \left(\left(\frac{M_O(1-\lambda)}{M_G(1-\phi)} + 1\right)A_T + A_P\right) \\ c = \frac{M_O}{(1-\phi)} \end{cases}$$

With initial condition $h(0) = 0$, $l(0) = 0$ ie. $dl/dt(0) = 0$. We write r_1 and r_2 as the roots of the characteristic equation.

l has the following form:

$$l(t) = \left(P_{physio}\frac{(1-\phi)}{M_O}\right) + K_1e^{r_1t} + K_2e^{r_2t}$$

h has the following form:

$$h(t) = P_{physio}\frac{(1-\phi)}{M_O} + K_1e^{r_1t}(1 + r_1\frac{A_T}{M_G}(1-\lambda)) + K_2e^{r_2t}(1 + r_2\frac{A_T}{M_G}(1-\lambda))$$

$[La]_m$ Kinetics

$$[La]_m(t) = 1/C_2[\mathbf{1}_{l<=\theta}l(t)A_T + \mathbf{1}_{l>\theta}((l-\theta)A_G + \theta A_T)]$$

In mmol/kg w. w. .

$\dot{V}O_2$ **Kinetics**

Therefore we can predict the $\dot{V}O_2$ evolution:

$$\dot{V}O_2 = 1/(\eta C_1)\left[P_{physio} + \frac{M_O}{(1-\phi)}\left(K_1 e^{r_1 t}(1+r_1\frac{A_T}{M_G}(1-\lambda)) + K_2 e^{r_2 t}(1+r_2\frac{A_T}{M_G}(1-\lambda))\right)\right]$$

In mLO_2/s.

Upper limit of the work rate region - LT1

Recalling equation Equation 1 with $dl/dt = 0$ and at equilibrium ($dh/dt = 0$) we have:

$$P_{physio} = \dot{V}O_{2,LT1}C_1 = M_O\left(\frac{h}{1-\phi}\right)$$

The largest possible value in this first case is reached when $h \longrightarrow \theta$. Therefore:

$$\boxed{\begin{cases} h_{LT1} = l_{LS1} = \theta \\ P_{LT1} = \eta P_{physio} = \eta M_O\left(\frac{\theta}{1-\phi}\right) \end{cases}} \quad (2)$$

Heavy work rate - Between LT1 and P_{crit}

Initially, $h < \theta$ and we will therefore observe the same behaviors as described previously until $h = \theta$ at a given time t_1. Then we have the following governing equations:

$$\begin{aligned} P_{physio} &= D_{O\to P} + \dot{V}_P + \dot{V}_G \\ &= M_O\left(\frac{h}{1-\phi}\right) + A_P\frac{dh}{dt} + A_G\frac{dl}{dt} \end{aligned}$$

With : $A_G\frac{dl}{dt} = M_G\left(\frac{h-l}{1-\lambda}\right)$

We can get a second-order differential equation on l or h. The one on l has the same form as the one for low work rate but A_T is replaced by A_G.

With initial conditions $l(t_1) = \theta$ and $dl/dt(t_1) = \frac{M_G}{A_G}\left(\frac{h-\theta}{1-\lambda}\right)$ we can similarly analytically solve the second-order differential equations.

$[La]_m$ **Kinetics**

$$[La]_m(t) = 1/C_2 l(t) A_G$$

In mmol/kg w. w. .

$\dot{V}O_2$ **Kinetics**

From h we can express the $\dot{V}O_2$ evolution:

$$\dot{V}O_2(t) = 1/C_1\left(P_{physio} + \frac{M_O}{(1-\phi)}l(t)\right)$$

In mLO_2/s.

Upper limit of the work rate region - P_{crit}^{meca}

P_{crit}^{meca} corresponds to the limit case in which we can still observe an equilibrium, ie. we do not reach exhaustion and satisfy the constrain. Recalling Equation 1 at equilibrium ($dh/dt = 0$ and $dl/dt = 0$, $h = l > \theta$). The largest possible value in this case is reached when $P_{physio} \longrightarrow P_{max}$.

$$P_{physio} = \dot{V}O_{2,P_{crit}}C_1 = M_O\left(\frac{h_{P_{crit}}}{1-\phi}\right)$$

$$P_{physio} = P_{max} = M_P\left(\frac{1-\lambda-l_{P_{crit}}}{1-\lambda-\theta}\right)$$

Therefore:

$$\boxed{\begin{cases} h_{P_{crit}} = l_{P_{crit}} = \frac{1-\lambda}{M_O/M_P(\frac{1-\theta-\lambda}{1-\phi})+1} \\ P_{crit}^{meca} = \eta C_1\dot{V}O_{2,P_{crit}} = \eta\frac{M_O(1-\lambda)}{(1-\phi)+M_O/M_P(1-\theta-\lambda)} \end{cases}} \quad (3)$$

"Severe work rate" - Exhaustion

The evolution will be the same as describe in previous case but no equilibrium will be reached i.e. the energy demand will be to high for it to be sustained by an increase in $\dot{V}O_2$ and we will reach exhaustion.

References

1. Morton, R. H. A three component model of human bioenergetics. *J. Math. Biol.* **24**, 451–466, DOI: 10.1007/BF01236892 (1986).
2. Morton, R. H. On a model of human bioenergetics II: Maximal power and endurance. *Eur. J. Appl. Physiol. Occup. Physiol.* **55**, 413–418, DOI: 10.1007/BF00422743 (1986).
3. Morton, R. Modelling human power and endurance. *J. Math. Biol.* **28**, DOI: 10.1007/BF00171518 (1990).
